# Age-Related Sex Disparities in Esophageal Cancer Survival: A Population-Based Study in the United States

**DOI:** 10.3389/fpubh.2022.836914

**Published:** 2022-07-12

**Authors:** Zhen-Fei Xiang, Hua-Cai Xiong, Dan-Fei Hu, Ming-Yao Li, Zhan-Chun Zhang, Zheng-Chun Mao, Er-Dong Shen

**Affiliations:** ^1^Department of Radiation Oncology, Ningbo Medical Center Lihuili Hospital, Ningbo University, Ningbo, China; ^2^Department of Thoracic Surgery, Ningbo Medical Center Lihuili Hospital, Ningbo University, Ningbo, China; ^3^Department of Oncology, Yueyang Central Hospital, Yueyang, China

**Keywords:** esophageal cancer, survival, SEER database, age-related, sex disparities

## Abstract

**Background:**

The association between sex and the survival of patients with esophageal cancer (EC) remains controversial. We sought to systematically investigate sex-based disparities in EC survival using the Surveillance, Epidemiology, and End Results (SEER) registry data from the United States.

**Methods:**

Patients with EC diagnosed from 2004 to 2015 registered in the SEER database were selected. The association between sex and cancer-specific survival (CSS) was evaluated using survival analysis. The Inverse Probability Weighting (IPW) approach was applied to reduce the observed bias between males and females. Subgroup analyses were used to investigate the robustness of the sex-based disparity and to explore potential interaction effects with other variables.

**Results:**

Overall, 29,312 eligible EC patients were analyzed, of whom 5,781 were females, and 23,531 were males. Females had higher crude CSS compared to males (10-year CSS: 24.5 vs. 21.3%; *P* < 0.001). Similar results were obtained after adjusting for selection bias using the IPW approach and multivariate regression. Subgroup analyses confirmed the relative robustness of sex as a prognostic factor. However, significant interactions were observed between sex and other variables, such as age, race, tumor grade, histology, and treatment modality. In particular, there was no survival advantage for premenopausal females compared to their male counterparts, but the association between sex and EC survival was prominent in 46–55-year-old patients.

**Conclusions:**

Female EC patients had better long-term survival than males. The association between sex and EC survival vary according to age, race, tumor grade, histology, and treatment modality. Sex-based disparity in EC-specific survival was age-related in the United States population.

## Introduction

Esophageal cancer (EC) is one of the most lethal gastrointestinal tumors, ranking sixth in cancer-related deaths. On a global scale, EC was responsible for one in every 18 cancer deaths in 2020 (1). EC is characterized by a unique geographical distribution, with the highest incidences recorded in East Asia and Southeast Africa and the lowest incidences in West Africa (2). The predominant histological types of EC are adenocarcinoma and squamous cell carcinoma. Historically, esophageal squamous cell carcinoma (ESCC) is the most prevalent pathological type. However, the incidence of esophageal adenocarcinoma (EAC) has increased in the last five decades. Esophageal adenocarcinoma has become the most common esophageal malignancy in western countries (3).

Male predominance is a significant epidemiological feature of EC. Globally, males have a 2.58-fold higher risk of EC incidence and a 2.59-fold higher risk of EC mortality than their female counterparts (1). The association between sex and EC survival has previously been examined in a series of studies under different circumstances (4–10). Previous studies have suggested a survival advantage for females compared with males, especially among younger women (4–6). Furthermore, Rowse et al. reported that women were correlated with improved complete pathologic response rates after neoadjuvant chemotherapy and higher recurrence-free survival rates after surgical intervention (7). However, some other studies failed to detect survival differences between males and females (8–10). Taken together, the association between sex and EC survival remains controversial.

Evidence from previous studies showed that sex-based disparities in EC survival could be partly attributed to sex-specific risk exposures, such as age at diagnosis, race, socioeconomic status, smoking, drinking, and histological types (2, 11, 12). However, sex-based disparities in cancer survival are sustained after adjusting for these known risk factors in many cases. In many studies, 55 years of age was generally used as a cut-off age to enter menopause because age is linked to changes in estrogen levels in the life cycle of females (5, 6). Mathieu et al. found that the annual percentage change in esophageal adenocarcinoma incidence rates for female patients during the same period was negatively correlated with ages 50–54 years and 60–64 years (13). Although the incidence of esophageal adenocarcinoma increased in male and female patients, the sex ratio across age peaked in ages 50–54 years and decreased thereafter. Insights from epidemiological studies and experimental research suggested that sex hormone may be an underlying mechanism that drives the survival advantage of females compared to males in patients with EC (4, 14, 15). Given the rationale mentioned above, the present study systematically examined the sex-based disparity in EC survival using the large-scale population obtained from the Surveillance, Epidemiology, and End Results (SEER) registry data in the United States.

## Patients and Methods

### Study Population

Research data were obtained from the SEER registry database after receiving approval for the use of data. We analyzed the database: the Incidence—SEER 18 Regs Custom Data (with additional treatment fields). We included data for EC cases diagnosed between 2004 and 2015, when the sixth edition of the American Joint Committee on Cancer (AJCC) staging system was accessible in the SEER program. Essential inclusion criteria were pathologically confirmed EC with available age, sex, race record, tumor grade, stage, and follow-up data. Any user can obtain the related data using SEER^*^Stat 8.3.8 software, given that they have been granted permission to access the SEER Program. Ethical approval was not necessary for this study with the publicly available database, as we had adhered to the terms for data usage.

### Study Variables and Primary Endpoint

We extracted demographic and clinicopathological characteristics from the SEER database directly using the SEER^*^Stat 8.3.8 software. The variables investigated were age at diagnosis, sex, tumor grade, histological classifications, disease stage, surgery recode, radiation recode, chemotherapy recodes, year of diagnosis, survival status, and survival time. Cancer-specific survival (CSS) was established as the primary endpoint in the current study. CSS was measured from the date of diagnosis of the disease to the date of death from EC. CSS was established as the primary end point, with the aim of reducing underlying bias caused by sex-based-specific differences other than the EC.

### Statistical Analysis

The Chi-square test was performed to examine the distributional differences in patient characteristics between male and female patients. CSS was estimated using the Kaplan-Meier approach. Survival differences were compared using the log-rank test. The association between sex and EC survival was evaluated using univariate and Inverse Probability Weighting (IPW)-adjusted Cox proportional hazards models. The IPW approach was applied to control observed differences in patient characteristics between males and females and then examined the exact association between sex and survival (16). In particular, IPWs were calculated based on propensity scores estimated from a logistic regression model using selected baseline variables. The calculation formula was as follows: $W\;=\;\frac{Z}{e}+\;\frac{1-Z}{1-e}$, where *Z* indicates sex (Z = 0 for male vs. Z = 1 for female), and *e* is the propensity score. We estimated the average association between sex and survival using IPWs, yielding a synthetic population where the observed baseline variables could not be confounded. Covariate balances before and after IPW were evaluated using absolute standardized differences (ASD). A difference of 0.05 or less represented an excellent balance. Further subgroup analyses were performed to investigate the robustness of the sex-based association and to examine potential interaction effects between sex and other variables, especially with age. Given that estrogen levels might play an essential role in mediating the impact of sex on EC survival. The associations between sex and EC survival were examined in different age subgroups, 18–45, 46–55, and >56 years, corresponding to premenopausal, perimenopausal, and postmenopausal periods, respectively, over the female lifecycle (17, 18). Finally, EAC and ESCC can actually be regarded as two very distinct diseases with different pathogenesis, epidemiology, and tumor biology. The robustness of age-related sex-based disparities on EC survival was also examined in EAC and ESCC, respectively.

All statistical analyses were performed using the R software (version 4.1.0). All tests were two-sided. A *P*-value of 0.05 or less was set as statistically significant.

## Results

### Patient Characteristics

**Figure 1:**
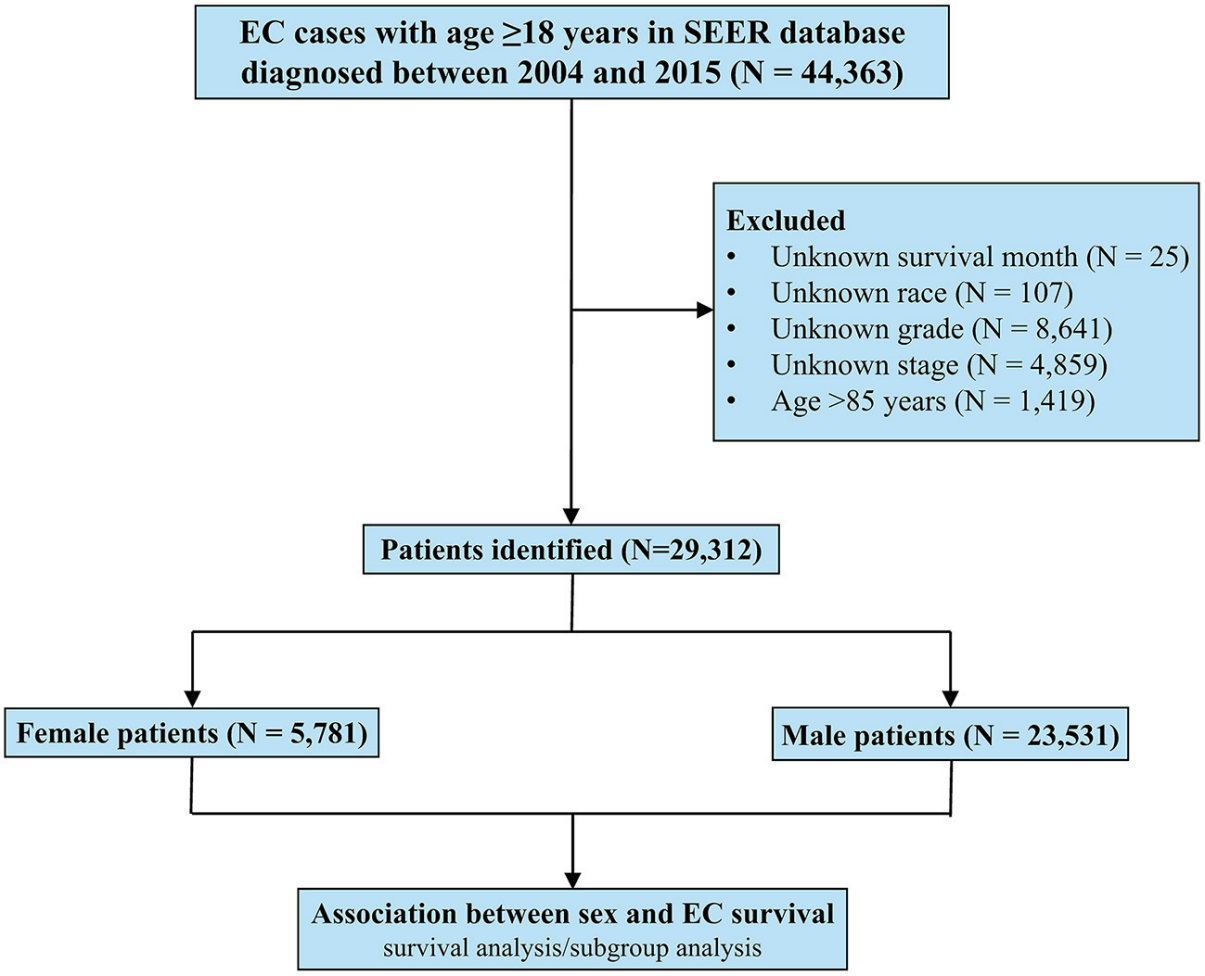
Patient selection flowchart.

This study included 29,312 EC patients diagnosed from 2004 to 2015 registered in the SEER database, of whom 5,781 were females and 23,531 were males (Figure 1). The patient characteristics stratified by sex are listed in Table 1. Female patients were significantly associated with older age (median age, 68 vs. 65 years, *P* < 0.001), black race (14.9 vs. 9.3%, *P* < 0.001), and squamous carcinoma (55.5 vs. 26.4%, *P* < 0.001). Male patients had higher proportions of worse differentiated tumors (54.5 vs. 49.0%, *P* < 0.001) and more advanced diseases (metastatic EC, 38.4 vs. 30.9%, *P* < 0.001), and were more likely to receive both surgery (31.3 vs. 26.3%, *P* < 0.001) and chemotherapy (66.6 vs. 61.9%, *P* < 0.001).

**Table 1:** Patient characteristics stratified by sex.

**Characteristics**	**Female**	**Male**	***P-*value**
	**(*N* = 5,781)**	**(*N* = 23,531)**	
Age (years)	68.0 (59.0–76.0)	65.0 (58.0–73.0)	<0.001
Race			<0.001
Black	862 (14.9)	2,192 (9.3)	
White	4,592 (79.4)	20,205 (85.9)	
Others	327 (5.7)	1,134 (4.8)	
Tumor grade			<0.001
Grade I	339 (5.9)	1,380 (5.9)	
Grade II	2,612 (45.2)	9,346 (39.7)	
Grade III	2,721 (47.1)	12,367 (52.6)	
Grade IV	109 (1.9)	438 (1.86)	
Histology			<0.001
Adenocarcinoma	2,314 (40.0)	16,263 (69.1)	
Squamous carcinoma	3,207 (55.5)	6,204 (26.4)	
Others	260 (4.5)	1,064 (4.5)	
Disease stage			<0.001
Localized EC	3,994 (69.1)	14,500 (61.6)	
Metastatic EC	1,787 (30.9)	9,031 (38.4)	
Surgery			<0.001
No or unknown	4,275 (73.9)	16,168 (68.7)	
Yes	1,506 (26.1)	7,363 (31.3)	
Radiation			0.149
No or unknown	2,356 (40.8)	9,838 (41.8)	
Yes	3,425 (59.2)	13,693 (58.2)	
Chemotherapy			<0.001
No or unknown	2,204 (38.1)	7,858 (33.4)	
Yes	3,577 (61.9)	15,673 (66.6)	
Year of diagnosis			0.044
2004–2007	1,896 (32.8)	7,317 (31.1)	
2008–2011	1,898 (32.8)	7,906 (33.6)	
2012–2015	1,987 (34.4)	8,308 (35.3)	

*EC, esophageal cancer*.

### Association Between Sex and EC Survival

**Figure 2:**
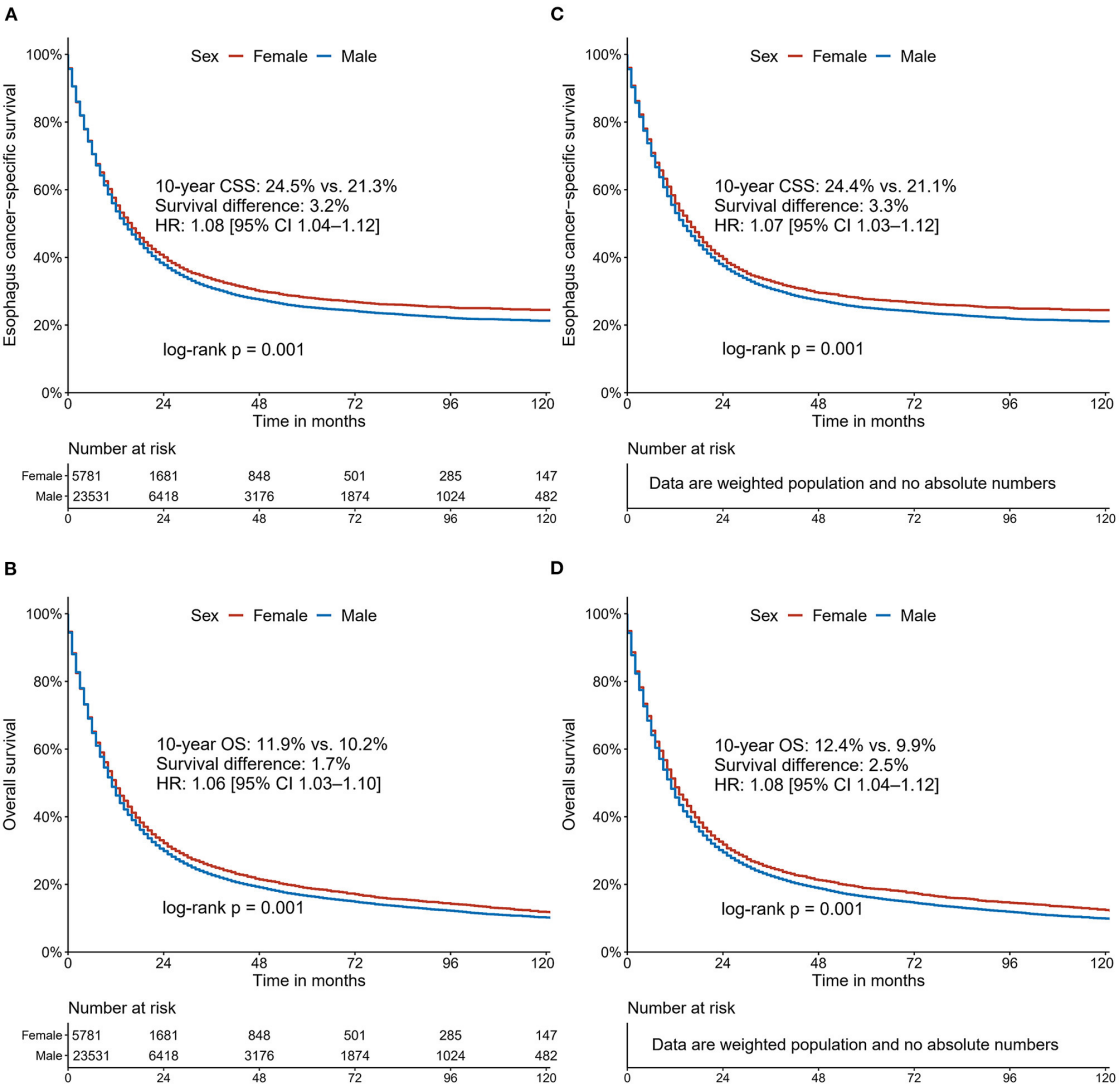
Cancer-specific survival and overall survival curves stratified by sex for patients with esophageal cancer: unadjusted **(A,B)**; Inverse Probability Weighting-adjusted **(C,D)**.

After a median follow-up of 75 [95% confidence interval (CI), 74–77] months, 3,594 deaths attributed to EC (62.2%) were recorded in females and 15,143 (64.4%) in males. In the unadjusted Kaplan-Meier analysis, female patients showed a higher CSS rate [10-year CSS: 24.5% (95% CI, 23.0–26.1) vs. 21.3% (95% CI, 20.5–22.0); HR: 1.06 (95% CI 1.02–1.10), *P* = 0.001; Figure 2A] and overall survival (OS) rate [10-year OS: 11.9% (95% CI, 10.8–13.1) vs. 10.2% (95% CI, 9.7–10.8); HR: 1.06 (95% CI 1.03–1.10), *P* < 0.001; Figure 2B] compared with male patients. Following IPW procedures, excellent balances were achieved between the two groups of sexes regarding all the patient characteristics examined (Supplementary Figure 1). IPW-adjusted Kaplan-Meier analyses also demonstrated a significantly better CSS rate [10-year CSS: 24.4% (95% CI, 22.8–26.2) vs. 21.1% (95% CI, 20.4–21.9); IPW-adjusted HR: 1.07 (95% CI 1.03–1.12), *P* < 0.001; Figure 2C] and OS rate [10-year OS: 24.4% (95% CI, 22.8–26.2) vs. 21.1% (95% CI, 20.4–21.9); and IPW-adjusted HR: 1.08 (95% CI 1.04–1.12), *P* < 0.001; Figure 2D] for female patients than for male patients. Further multivariate analyses revealed that sex was identified as an independent prognostic factor for CSS [HR: 1.14 (95% CI 1.09–1.18), *P* < 0.001] and OS [HR: 1.16 (95% CI 1.12–1.20), *P* < 0.001] in multivariate Cox regression analysis after adjustment for demographic, clinicopathological, and therapeutic factors.

### Subgroup Analyses and Interaction Effects

**Figure 3:**
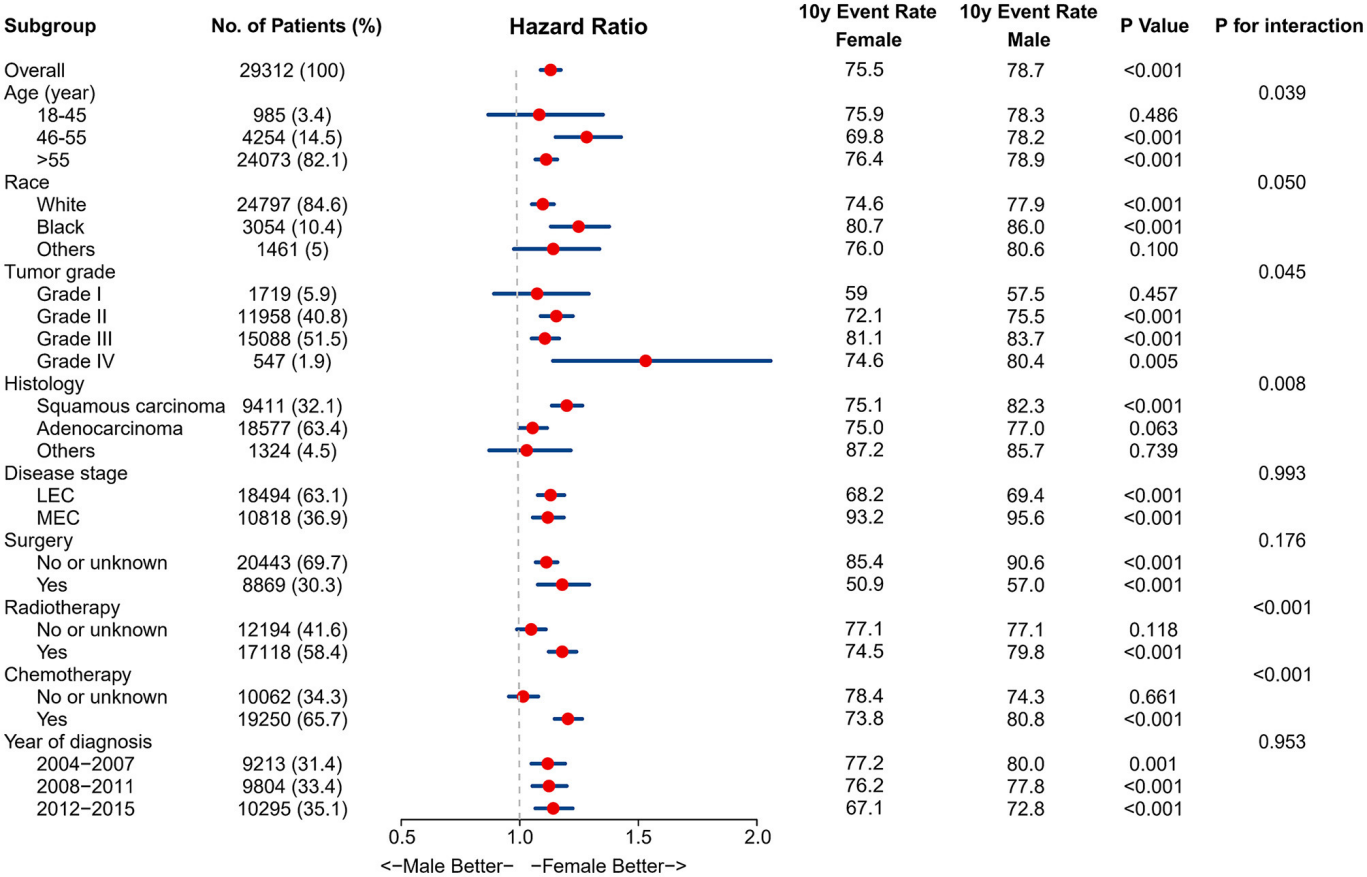
Results for the subgroup analyses and interaction tests of sex-based effects on cancer-specific survival are summarized in a Forest plot.

The association between sex and EC survival was further explored in subgroups defined by age, race, tumor grade, histology, disease stage, treatment modalities, and year of diagnosis. The results of the CSS subgroup analyses are displayed in a Forest plot (Figure 3). The forest plot confirmed the relative robustness of sex as a prognostic factor in many subgroups. However, some significant interactions were observed between sex and other variables on EC survival, such as age, race, tumor grade, histology, radiotherapy, and chemotherapy. The association between sex and EC survival was more marked in patients aged 46–55 years, black and white race, advanced differentiated or squamous carcinoma, and patients who received radiotherapy and chemotherapy.

### Age-Dependent Association Between Sex and EC Survival

**Figure 4:**
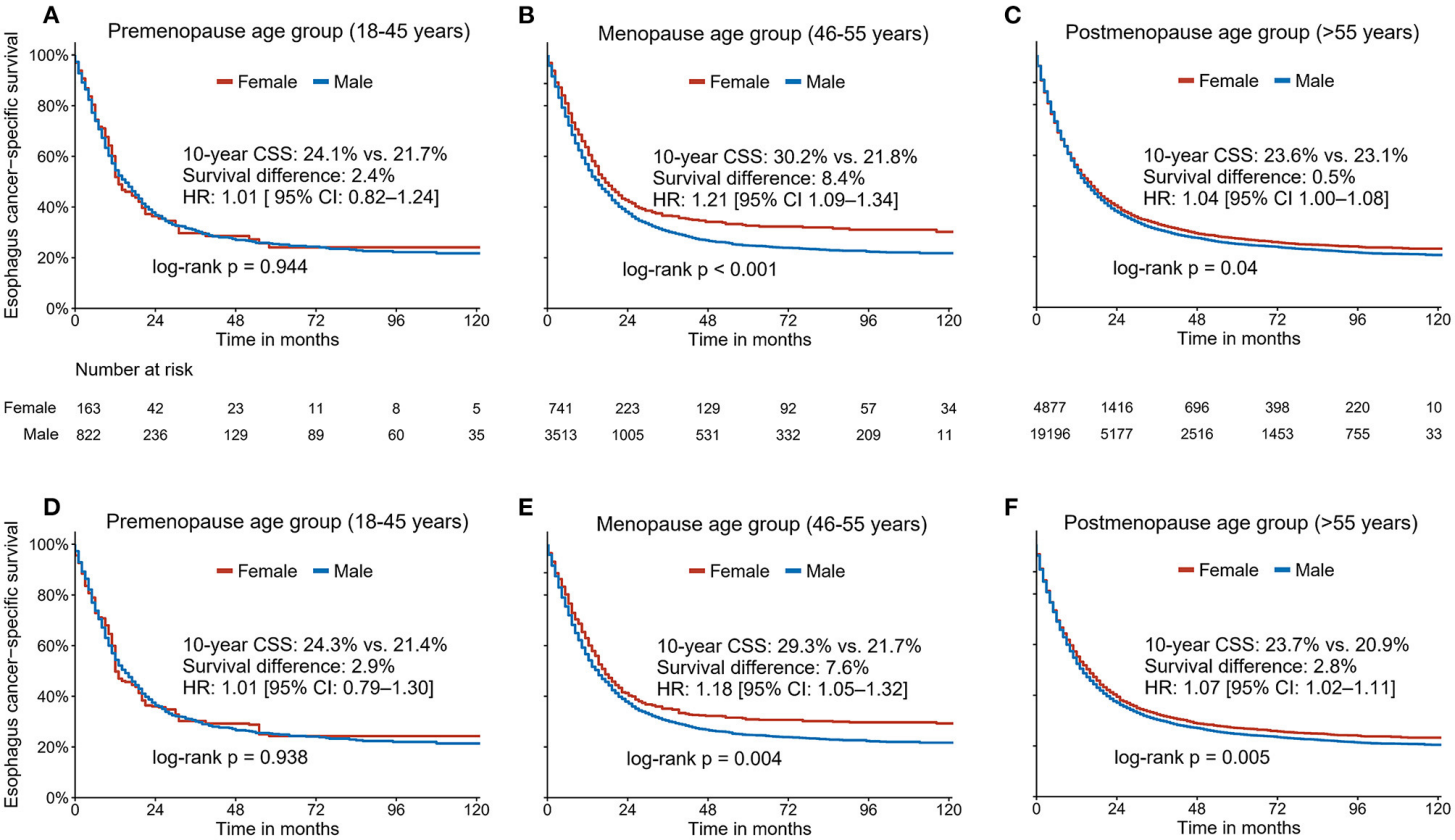
The unadjusted **(A–C)** and Inverse Probability Weighting-adjusted **(D–F)** association between sex and cancer-specific survival in three different age groups (premenopausal age group: age ≤ 45 years; perimenopause age group: ages 46–55; and postmenopausal age group: age ≥ 56 years).

We further examined the association between sex and EC survival in different age subgroups. The patient characteristics stratified by sex across different age groups are summarized in Supplementary Tables 1–3. We observed a significant interaction between age and sex (*P* for interaction <0.05). Kaplan-Meier analyses without adjustment did not show a survival advantage for premenopausal females compared to their male counterparts [10-year CSS: 24.1% (95% CI, 17.2–33.8) vs. 21.7% (95% CI, 18.5–25.6); HR 1.01, 95% CI 0.82–1.24; *P* = 0.944; Figure 4A]. Notably, the survival advantage associated with females was most evident in perimenopausal patients [10-year CSS: 30.2% (95% CI, 26.2–34.8) vs. 21.8% (95% CI, 20.1–23.6); HR 1.21, 95% CI 1.09–1.34; *P* < 0.001; Figure 4B]. However, such sex benefits declined in postmenopausal patients [10-year CSS: 23.6% (95% CI, 22.0–25.3) vs. 23.1% (95% CI, 20.2–22.0); HR 1.04, 95% CI 1.00–1.08; *P* = 0.04; Figure 4C]. Similarly, the IPW approach was applied to control for the observed bias, resulting in well-balanced samples (Supplementary Figures 2–4). The IPW-adjusted Kaplan-Meier analyses yielded comparable results as mentioned above (Figures 4D–F).

### Histology-Based Age-Related Sex-Based Disparities in EC Survival

Given that EAC and ESCC are two very distinct diseases with different pathogenesis, epidemiology and tumor biology. The robustness of age-related sex-based disparities on EC survival was also examined for EAC and ESCC, respectively. The same statistical strategies were used to control the observed bias (Supplementary Figures 5, 6). We found that women were associated with worse CSS and OS compared to men, regardless of histology (Supplementary Figures 7, 8). In the subgroup analysis that evaluated age-related sex-based disparities in different histology subtypes, similar age-dependent sex-based disparities in the total population were observed. That is, the survival advantage associated with females was not significant in the premenopausal period (Supplementary Figures 9A,B, 10A,B), but was marked in the perimenopausal period (Supplementary Figures 9C,D, 10C,D) and declined in the postmenopausal period (Supplementary Figures 9E,F, 10E,F).

## Discussion

Mass data from the SEER 18 Registry database allowed us to investigate the sex-based disparity in EC survival in multiple dimensions. To our knowledge, this is one of the most extensive cohort studies to examine the sex-based disparity in EC. We demonstrated a significant survival advantage of females over males in patients with EC. Sex was identified as an independent prognostic factor. Sex-based disparities in EC survival were significantly correlated with age, race, tumor grade, histology, and therapeutic pattern. In particular, sex-based disparities in EC-specific survival appeared to be age dependent.

Over the past few decades, the association between sex and EC survival have been studied. Most of the previous studies focused on studying trends and prognosis (3, 9, 19, 20). The potential interaction with underlying confounding factors has not been fully considered when describing differences in survival based on sex. It has been frequently assumed that sex-based differences are constant throughout the life cycle. Other shortcomings include inadequate follow-up, potential selection bias, small sample size, and insufficient bias adjustment. Due to different research settings, these problems will increase the complexity and uncertainty when investigating the association between sex and EC survival. Given the lack of convincing evidence, we examined the association between sex and EC survival using an extensive patient sample with sufficient follow-up duration. The IPW method was used to minimize the underlying confusion and selection bias. At the same time, the interaction of other factors on sex-based survival differences has been studied, and the age-related associations of sex on mortality attributed to EC has been further evaluated.

In previous studies, there has been controversy about the association between sex and the survival of patients with EC. Our findings are in line with the results of several previous studies. Some retrospective series have shown that sex is significantly associated with the prognosis of EC (3, 9, 19, 20). Wang et al. reported that females diagnosed with EC had a significantly prolonged 5-year survival rate than males (female vs. male: 48.2 vs. 28.7%, *P* = 0.003) (21). Similarly, Bohanes et al. demonstrated that women had a more prolonged EC-specific survival than men, regardless of metastatic EC and localized EC cohorts (5). However, another study that included 1,718 Chinese and 1,624 white ESCC patients came to the opposite conclusion (8). Sex was no longer an independent prognostic factor in Chinese and white patients living in the United States (8). In this study, we found that sex was an independent prognostic factor in patients with EC. Females showed higher CSS rates than males, even after adequately adjusting for confounding factors. Furthermore, subgroup analysis confirmed the relative robustness of sex as a prognostic factor.

Interestingly, we found that sex-based differences in survival interacted with age, race, tumor grade, histology, and treatment pattern. The association of sex-based differences and survival was even more profound in patients aged 46–55 years, black and white people, advanced differentiated or squamous carcinoma, and patients who received radiation therapy and chemotherapy. This finding may partially explain the discrepancy in the results of previous studies. Differences in the study population, age structure, and ethnic composition may lead to heterogeneous research results. In this study, we applied the IPW method to reduce the influence of these confounders when examining the association between sex and EC survival, increasing the credibility of our findings.

Our subgroup analysis detected a significant interaction effect between age and sex (*P* < 0.05). In addition, we explored the age-dependent relationship between sex and EC survival. Several previous studies have suggested that intrinsic biological sex hormones could be an underlying mechanism that explains the female advantage in cancer incidence and morbidity (6, 8, 9, 14, 22). Age 46–55 years is generally used as a surrogate for the perimenopausal period. Many studies divide patients into different age groups as a substitute for changes in sex hormone levels (5). Su et al. reported that females younger than 55 years had a reduced risk of dying of ESCC than males of similar age and women and men 55 years or older. Their results suggested that sex hormones may have a protective effect in female patients with EC (6). However, Bohanes et al. failed to explain the sex-based difference in EC survival attributed to hormone protection (5). They reported that females under 55 years of age and those 55 years or older with squamous cell LEC had better ECSS than men. However, only females under 55 years of age had longer ECSS than men in the metastatic squamous cell EC population. The results of the metastatic EC cohort are consistent with the phenomenon of sex hormone protection. Moreover, using the results of a localized EC cohort it is difficult to provide convincing evidence of sex hormone protection because sex-based differences of individuals aged over 55 years old seem to be more pronounced.

In this study, we divided the patients into three age subgroups: 18–45 years, 46–55 years, and >56 years old, corresponding to the premenopausal, perimenopausal, and postmenopausal periods of women's lifecycle, respectively, and further studied the relationship between sex and EC survival in these periods. Survival analysis showed that premenopausal women (18–45 years) did not have a survival advantage compared to men, and the female-associated survival advantage was evident in menopausal patients (46–55 years). However, this sex-based advantage declined in postmenopausal patients (>55 years). The findings were similar even after adjusting for many clinical parameters. Our results cannot explain the differences based on sex in EC survival possibly due to sex hormone protection. Some studies have reported that the estrogen level of menopausal women is significantly higher than that of premenopausal women, which might explain the apparent phenomenon of a female-related survival advantage in menopausal (46–55 years old) patients (23, 24). It is still challenging to use hormone protection to explain the disappearing survival advantage in premenopausal (18–45 years) and postmenopausal (>55 years) patients. Therefore, we believe that it may be inappropriate to solely explain sex-based differences in EC survival with changes in sex hormone levels. Future studies are needed to reveal the underlying mechanisms for this phenomenon. However, our results correct one misleading conclusion: It may not be appropriate to assume that sex-based differences persist throughout life. Except for sex hormones, the overall survival difference between male and female patients suggests that unexplained mediation factors may explain the age-dependent sex-based disparity in EC survival (25).

This study has some inevitable limitations that must be noted. First, the data were drawn from the SEER registry database. Detailed information on family income, medical insurance, education, smoking, and alcohol consumption is missing and was not considered in our analyses, which could differ between males and females. The above socioeconomic variables could play a role in the survival of EC patients (5, 12, 26–28). Potential interactions between sex and these socioeconomic variables should be considered if possible. Second, due to the nature of the retrospective design and registry database, data on serum sex hormone levels were unavailable. Using age as a surrogate for menopause may raise confounding bias. Third, the study population derived from the United States, and external validation of the results from other countries will enhance our findings. Lastly, though we have detected statistical significances, the absolute survival differences between males and females were not large in the entire cohort. Besides, we did not examine to what degree the sex-based disparities in the survival of EC could be explained by mediating or confounding effects.

In conclusion, female patients with EC had better CSS than their male counterparts. This sex-based disparity interacted with age, race, tumor grade, histology, and therapeutic pattern. Sex-based disparity in EC-specific survival was age-related in the United States population. Future studies should continue to explore the potential causes of sex-based differences in survival in patients with EC.

## Data Availability Statement

The raw data supporting the conclusions of this article will be made available by the authors, without undue reservation.

## Ethics Statement

Ethical review and approval was not required for the study on human participants in accordance with the local legislation and institutional requirements. Written informed consent for participation was not required for this study in accordance with the national legislation and the institutional requirements.

## Author Contributions

E-DS, Z-CM, and Z-FX designed the study and participated in the acquisition of data. Z-FX developed the methodology of the study, analyzed and interpreted the data, and wrote the manuscript. All authors reviewed and revised the manuscript. All authors contributed to the article and approved the submitted version.

## Funding

This study was supported by the Medical Science and Technology Project of Zhejiang Provincial Health Commission (2019KY188), the Natural Science Foundation of Ningbo (2021J289), the Ningbo Clinical Research Center for Thoracic and Breast Neoplasms (2021L002), and the Major Science and Technology Innovation in 2025 Projects of Ningbo, China (2019B10039).

## Conflict of Interest

The authors declare that the research was conducted in the absence of any commercial or financial relationships that could be construed as a potential conflict of interest.

## Publisher's Note

All claims expressed in this article are solely those of the authors and do not necessarily represent those of their affiliated organizations, or those of the publisher, the editors and the reviewers. Any product that may be evaluated in this article, or claim that may be made by its manufacturer, is not guaranteed or endorsed by the publisher.
