# Supplementary material for: Age-Related Sex Disparities in Esophageal Cancer Survival: A Population-Based Study in the United States
Source: Front Public Health. 2022 Jul 12;10:836914. doi: 10.3389/fpubh.2022.836914 (PMC9314568; doi:10.3389/fpubh.2022.836914)
Supplement: Supplementary file 1 [file Data_Sheet_1.PDF]

frontiers

Supplementary Material

1 Supplementary Figures and Tables

Table S1. Patient characteristics stratified by sex in patients age 18-45 years.

Table S2. Patient characteristics stratified by sex in patients age 46-55 years.

Table S3. Patient characteristics stratified by sex in patients age >55 years.

Figure S1. Covariate balance between sex groups before and after the Inverse Probability Weighting (IPW).

Figure S2. Covariate balance between sex groups among patients aged 18-45 years before and after the Inverse Probability Weighting (IPW).

Figure S3. Covariate balance between sex groups among patients aged 46-55 years before and after the Inverse Probability Weighting (IPW).

Figure S4. Covariate balance between sex groups among patients aged >55 years before and after the Inverse Probability Weighting (IPW).

Figure S5. Covariate balance between sex groups before and after the Inverse Probability Weighting (IPW) among esophageal adenocarcinoma (EAC) patients (A), EAC patients aged 18-45 years (B), EAC patients aged 46-55 years (C), and EAC patients aged >55 years (D).

Figure S6. Covariate balance between sex groups before and after the Inverse Probability Weighting (IPW) among esophageal squamous cell carcinoma (ESCC) patients (A), ESCC patients aged 18-45 years (B), ESCC patients aged 46-55 years (C), and ESCC patients aged >55 years (D).

Figure S7. Cancer-specific survival and overall survival curves by sex for patients with esophageal adenocarcinoma: (A and B) unadjusted; (C and D) Inverse Probability Weighting-adjusted.

Figure S8. Cancer-specific survival and overall survival curves by sex for patients with esophageal squamous cell carcinoma: (A and B) unadjusted; (C and D) Inverse Probability Weighting-adjusted.

Figure S9. The unadjusted (A, B, C) and Inverse Probability Weighting-adjusted (D, E, F) association between sex and cancer-specific survival in three different age group among patients with esophageal adenocarcinoma (premenopausal age group: age 45 or less; perimenopause age group: age 46-55; and postmenopausal age group: age 56 or more).

Figure S10. The unadjusted (A, B, C) and Inverse Probability Weighting-adjusted (D, E, F) association between sex and cancer-specific survival in three different age group among patients with esophageal squamous cell carcinoma (premenopausal age group: age 45 or less; perimenopause age group: age 46-55; and postmenopausal age group: age 56 or more).

Table S1. Patient characteristics stratified by sex in patients aged 18-45 years.

Characteristics	Female (N=163)	Male (N=822)	P value
Age (years)	42.0 [38.5;44.0]	42.0 [39.0;44.0]	0.480
Race			0.005
Black	32 (19.6)	88 (10.7)	
Others	11 (6.8)	51 (6.2)	
White	120 (73.6)	683 (83.1)	
Tumor Grade			0.633
Grade I	15 (9.20)	54 (6.6)	
Grade II	62 (38.0)	332 (40.4)	
Grade III	82 (50.3)	417 (50.7)	
Grade IV	4 (2.5)	19 (2.3)	
Histology			<0.001
Adenocarcinoma	84 (51.5)	630 (76.6)	
Others	6 (3.7)	47 (5.7)	
Squamous carcinoma	73 (44.8)	145 (17.6)	
Disease stage			0.083
LEC	95 (58.3)	415 (50.5)	
MEC	68 (41.7)	407 (49.5)	
Surgery			0.038
No or unknown	120 (73.6)	533 (64.8)	
Yes	43 (26.4)	289 (35.2)	
Radiation			0.370
No or unknown	64 (39.3)	357 (43.4)	
Yes	99 (60.7)	465 (56.6)	
Chemotherapy			0.852
No or unknown	35 (21.5)	185 (22.5)	
Yes	128 (78.5)	637 (77.5)	
Year of diagnosis			0.104
2004-2007	51 (31.3)	300 (36.5)	
2008-2011	64 (39.3)	253 (30.8)	
2012-2015	48 (29.4)	269 (32.7)	

Abbreviation, LEC: localized esophagus cancer; MEC, metastatic esophagus cancer.

Table S2. Patient characteristics stratified by sex in patients aged 46-55 years.

Characteristics	Female (N=741)	Male (N=3,513)	P value
Age (years)	52.0 [50.0;54.0]	52.0 [49.0;54.0]	0.353
Race			<0.001
Black	170 (22.9)	434 (12.4)	
Others	33 (4.5)	193 (5.5)	
White	538 (72.6)	2886 (82.2)	
Tumor Grade			0.024
Grade I	45 (6.07)	217 (6.18)	
Grade II	341 (46.0)	1410 (40.1)	
Grade III	344 (46.4)	1814 (51.6)	
Grade IV	11 (1.5)	72 (2.1)	
Histology			<0.001
Adenocarcinoma	308 (41.6)	2448 (69.7)	
Others	21 (2.8)	157 (4.47)	
Squamous carcinoma	412 (55.6)	908 (25.8)	
Disease stage			0.001
LEC	462 (62.3)	1949 (55.5)	
MEC	279 (37.7)	1564 (44.5)	
Surgery			0.466
No or unknown	498 (67.2)	2309 (65.7)	
Yes	243 (32.8)	1204 (34.3)	
Radiation			0.029
No or unknown	267 (36.0)	1420 (40.4)	
Yes	474 (64.0)	2093 (59.6)	
Chemotherapy			0.786
No or unknown	201 (27.1)	933 (26.6)	
Yes	540 (72.9)	2580 (73.4)	
Year of diagnosis			0.610
2004-2007	240 (32.4)	1181 (33.6)	
2008-2011	258 (34.8)	1244 (35.4)	
2012-2015	243 (32.8)	1088 (31.0)	

Abbreviation: LEC, localized esophagus cancer; MEC, metastatic esophagus cancer.

Table S3. Patient characteristics stratified by sex in patients aged >55 years.

Characteristics	Female (N=4,877)	Male (N=19,196)	P value
Age (years)	71.0 [64.0;77.0]	68.0 [62.0;75.0]	<0.001
Race			<0.001
Black	660 (13.5)	1670 (8.70)	
Others	283 (5.80)	890 (4.64)	
White	3934 (80.7)	16636 (86.7)	
Tumor Grade			<0.001
Grade I	279 (5.7)	1109 (5.78)	
Grade II	2209 (45.3)	7604 (39.6)	
Grade III	2295 (47.1)	10136 (52.8)	
Grade IV	94 (1.9)	347 (1.81)	
Histology			0.000
Adenocarcinoma	1922 (39.4)	13185 (68.7)	
Others	233 (4.8)	860 (4.5)	
Squamous carcinoma	2722 (55.8)	5151 (26.8)	
Disease stage			<0.001
LEC	3437 (70.5)	12136 (63.2)	
MEC	1440 (29.5)	7060 (36.8)	
Surgery			<0.001
No or unknown	3657 (75.0)	13326 (69.4)	
Yes	1220 (25.0)	5870 (30.6)	
Radiation			0.562
No or unknown	2025 (41.5)	8061 (42.0)	
Yes	2852 (58.5)	11135 (58.0)	
Chemotherapy			<0.001
No or unknown	1968 (40.4)	6740 (35.1)	
Yes	2909 (59.6)	12456 (64.9)	
Year of diagnosis			0.003
2004-2007	1605 (32.9)	5836 (30.4)	
2008-2011	1576 (32.3)	6409 (33.4)	
2012-2015	1696 (34.8)	6951 (36.2)	

Abbreviation: LEC, localized esophagus cancer; MEC, metastatic esophagus cancer.

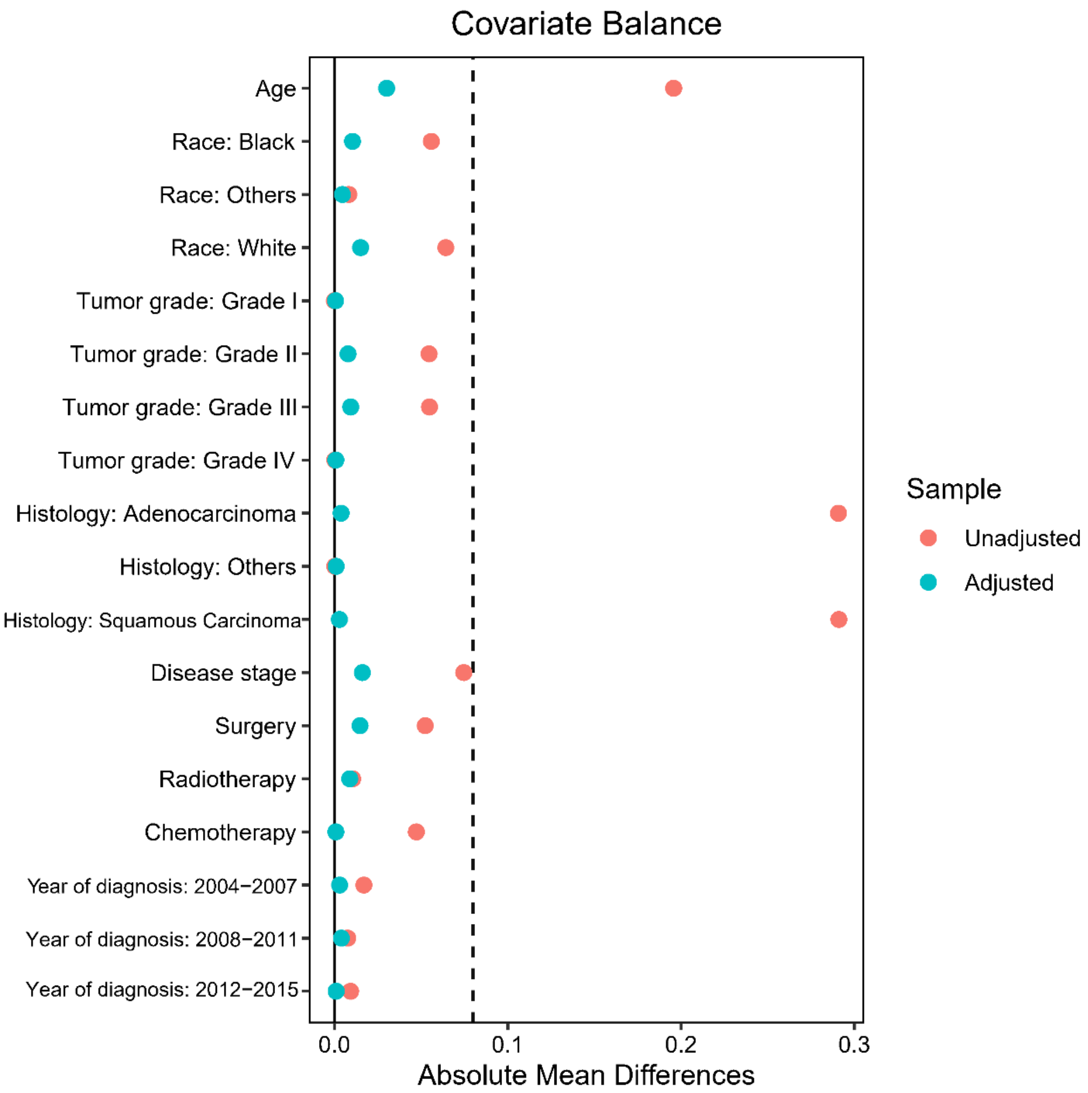

Figure S1. Covariate balance between sex groups before and after the inverse probability of treatment weighted (IPW).

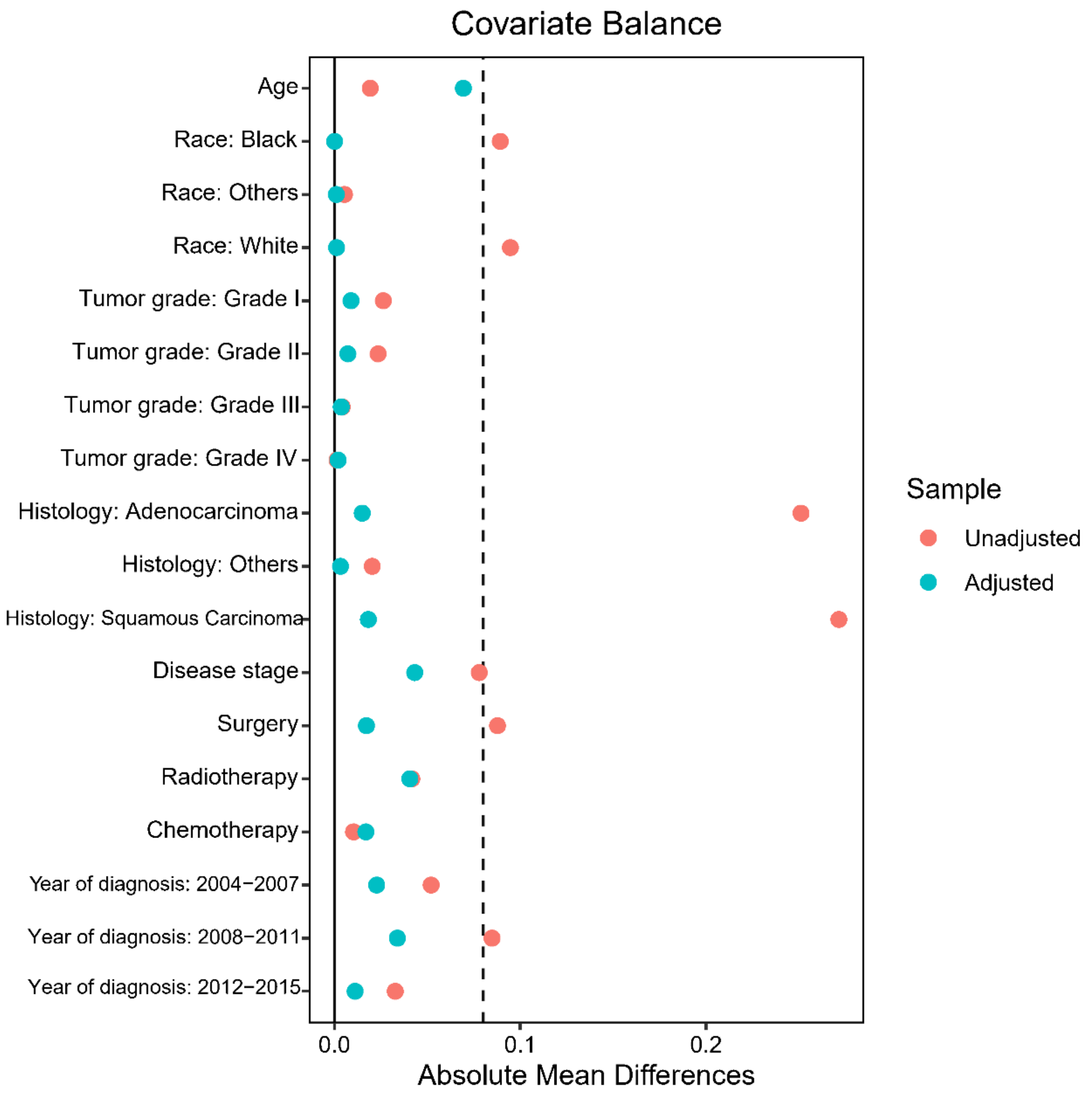

Figure S2. Covariate balance between sex groups among patients aged 18-45 years before and after the inverse probability of treatment weighted (IPW).

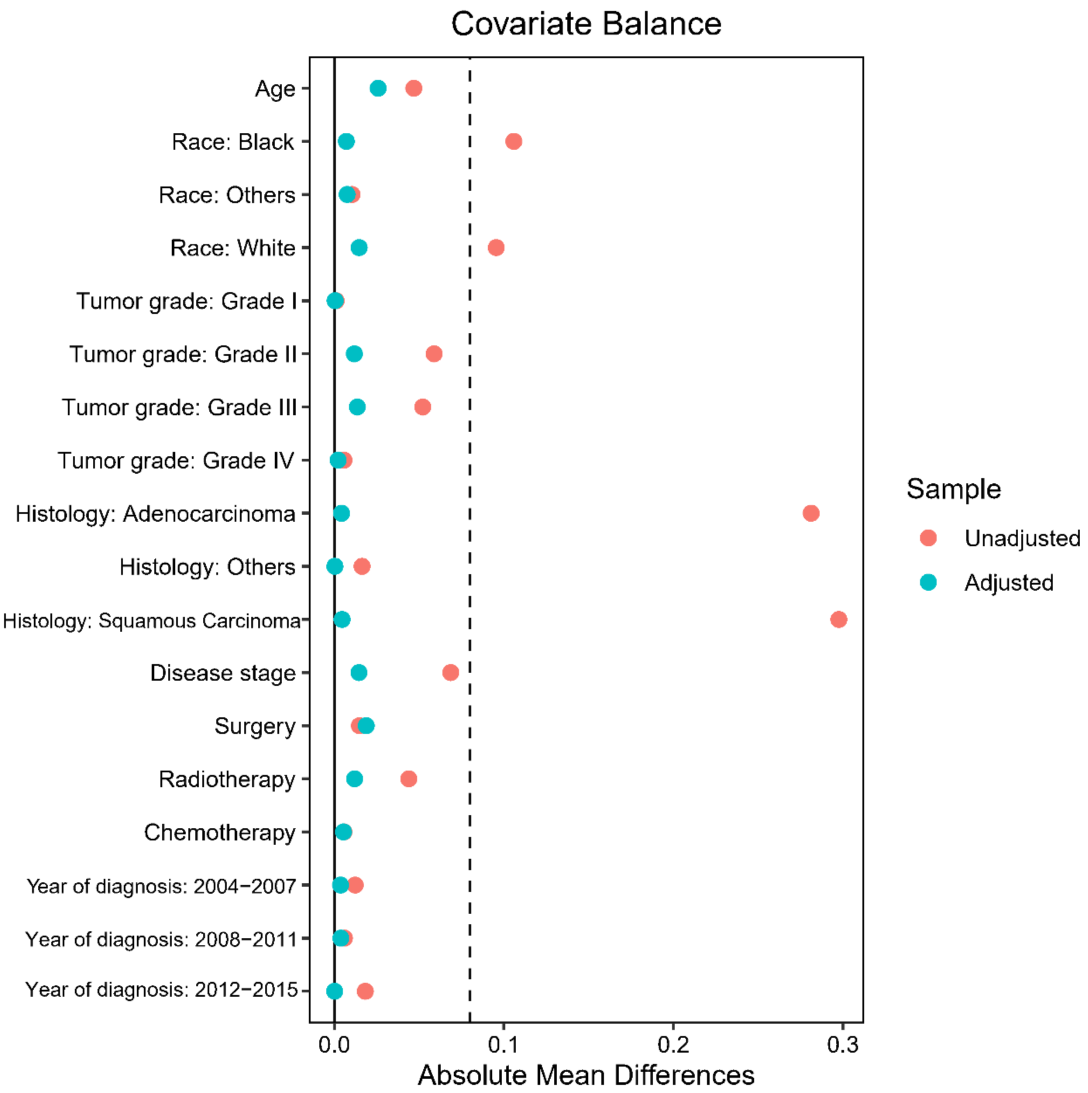

Figure S3. Covariate balance between sex groups among patients aged 46-55 years before and after the inverse probability of treatment weighted (IPW).

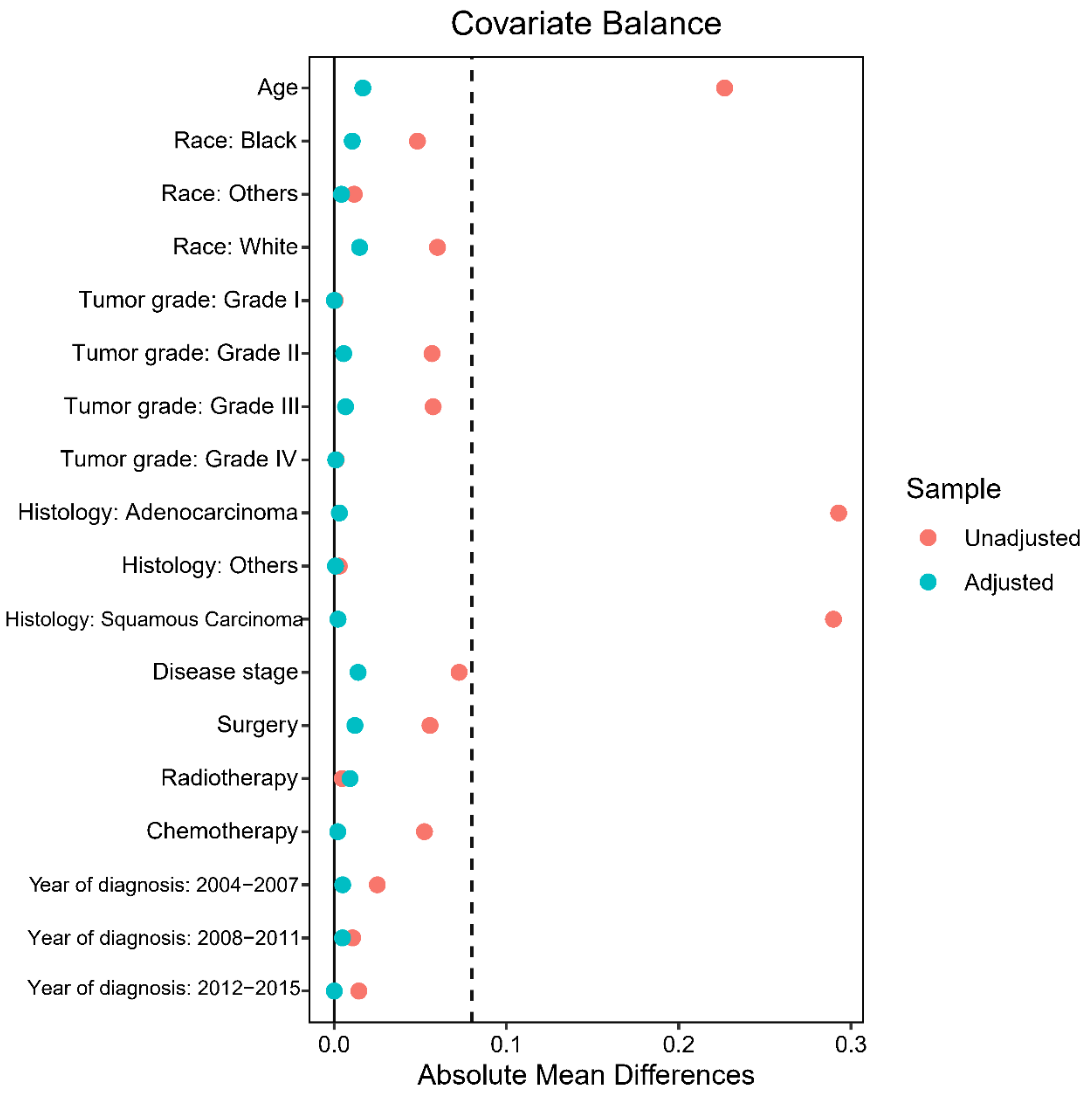

Figure S4. Covariate balance between sex groups among patients aged >55 years before and after the inverse probability of treatment weighted (IPW).

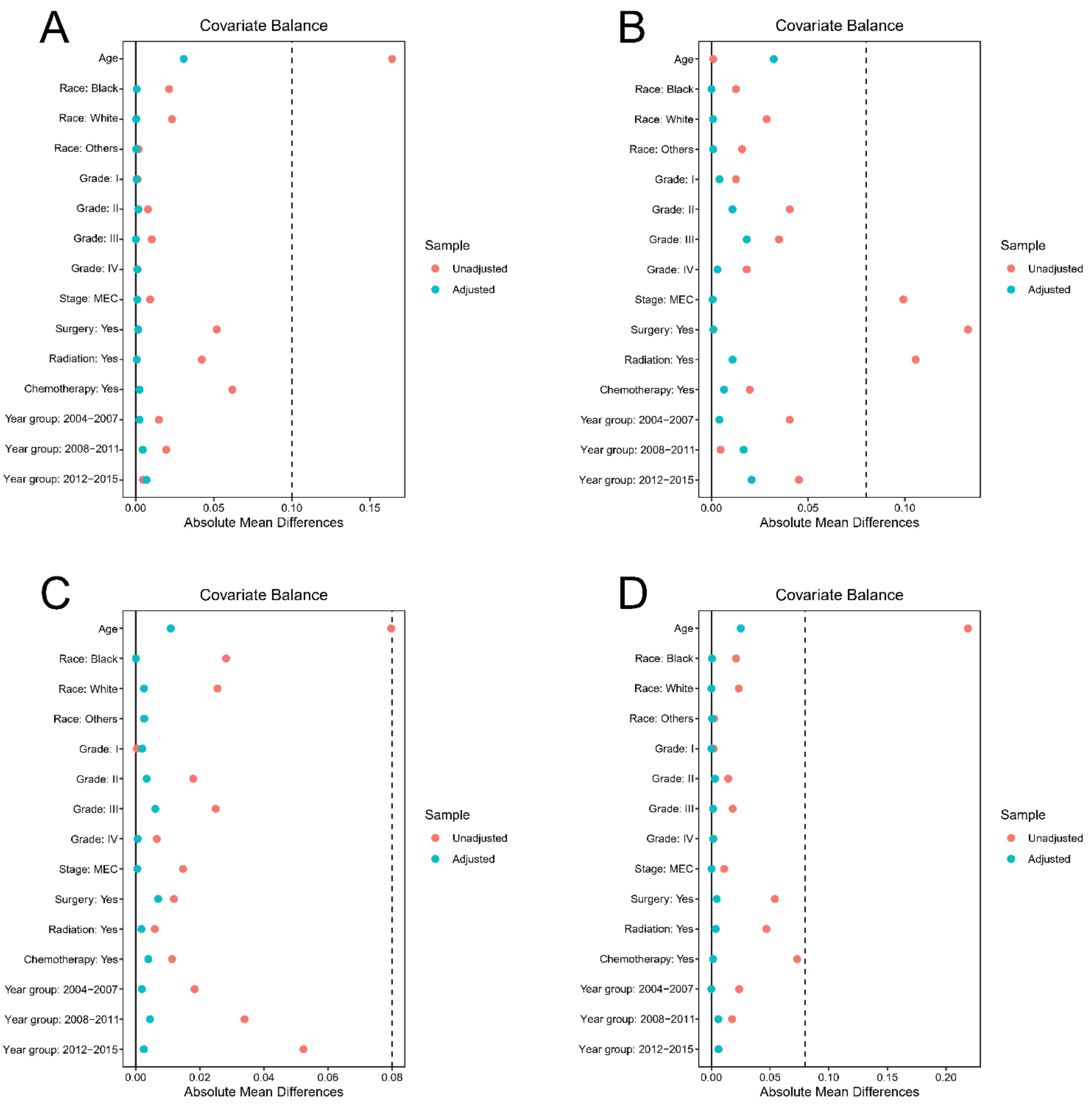

Figure S5. Covariate balance between sex groups before and after the Inverse Probability Weighting (IPW) among esophageal adenocarcinoma (EAC) patients (A), EAC patients aged 18-45 years (B), EAC patients aged 46-55 years (C), and EAC patients aged >55 years (D).

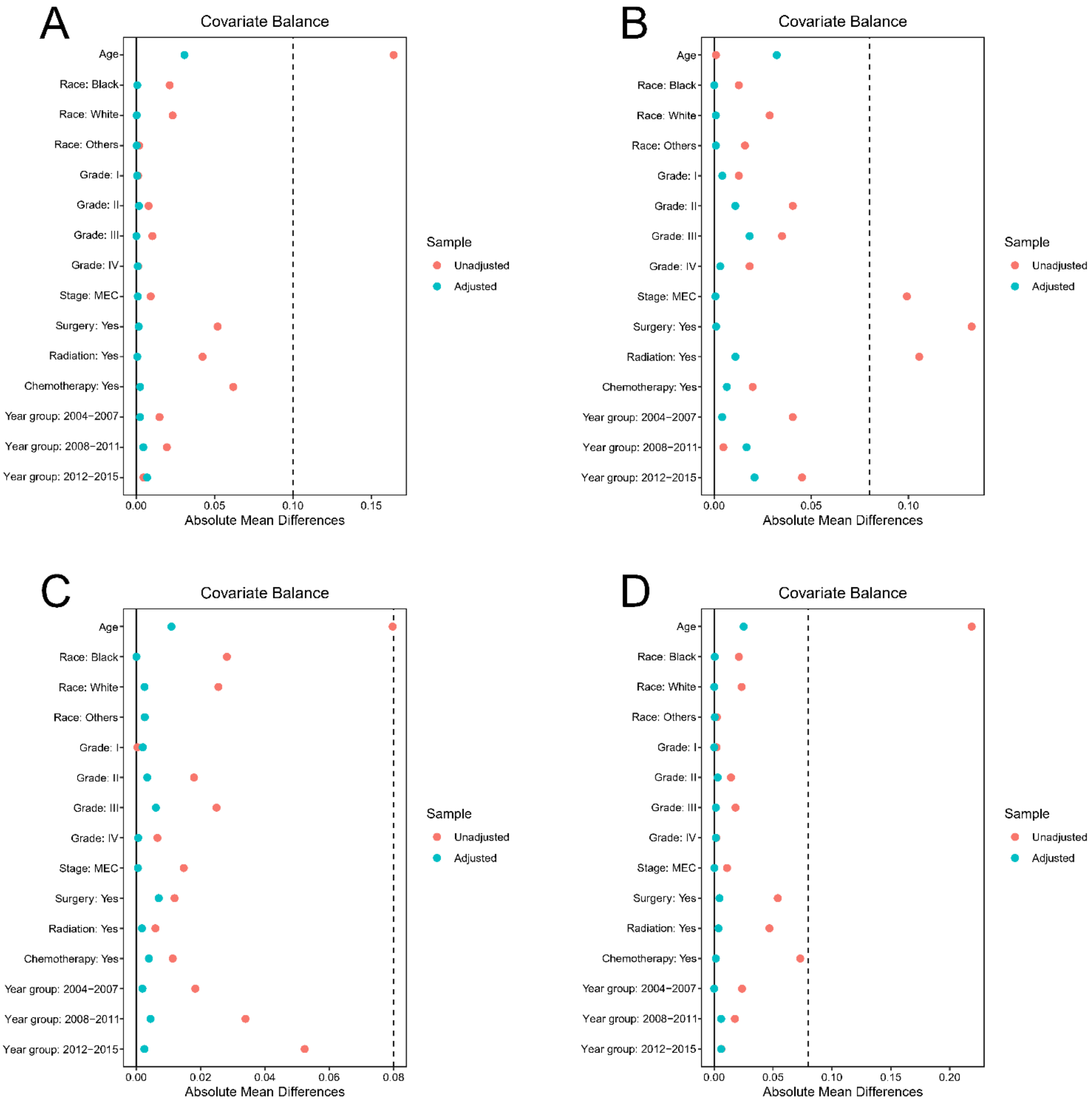

Figure S6. Covariate balance between sex groups before and after the Inverse Probability Weighting (IPW) among esophageal squamous cell carcinoma (ESCC) patients (A), ESCC patients aged 18-45 years (B), ESCC patients aged 46-55 years (C), and ESCC patients aged >55 years (D).

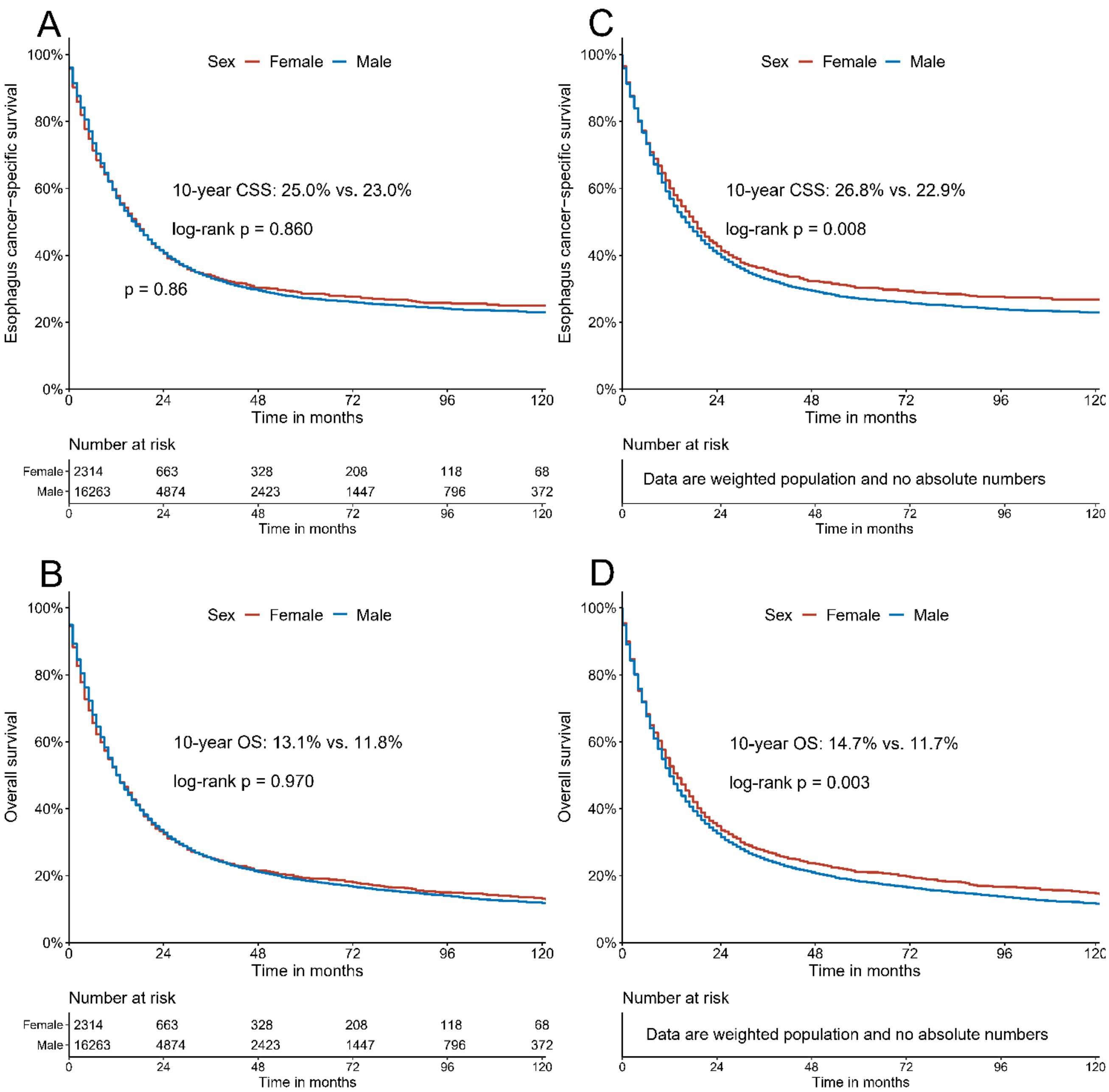

Figure S7. Cancer-specific survival and overall survival curves by sex for patients with esophageal adenocarcinoma: (A and B) unadjusted; (C and D) Inverse Probability Weighting-adjusted.

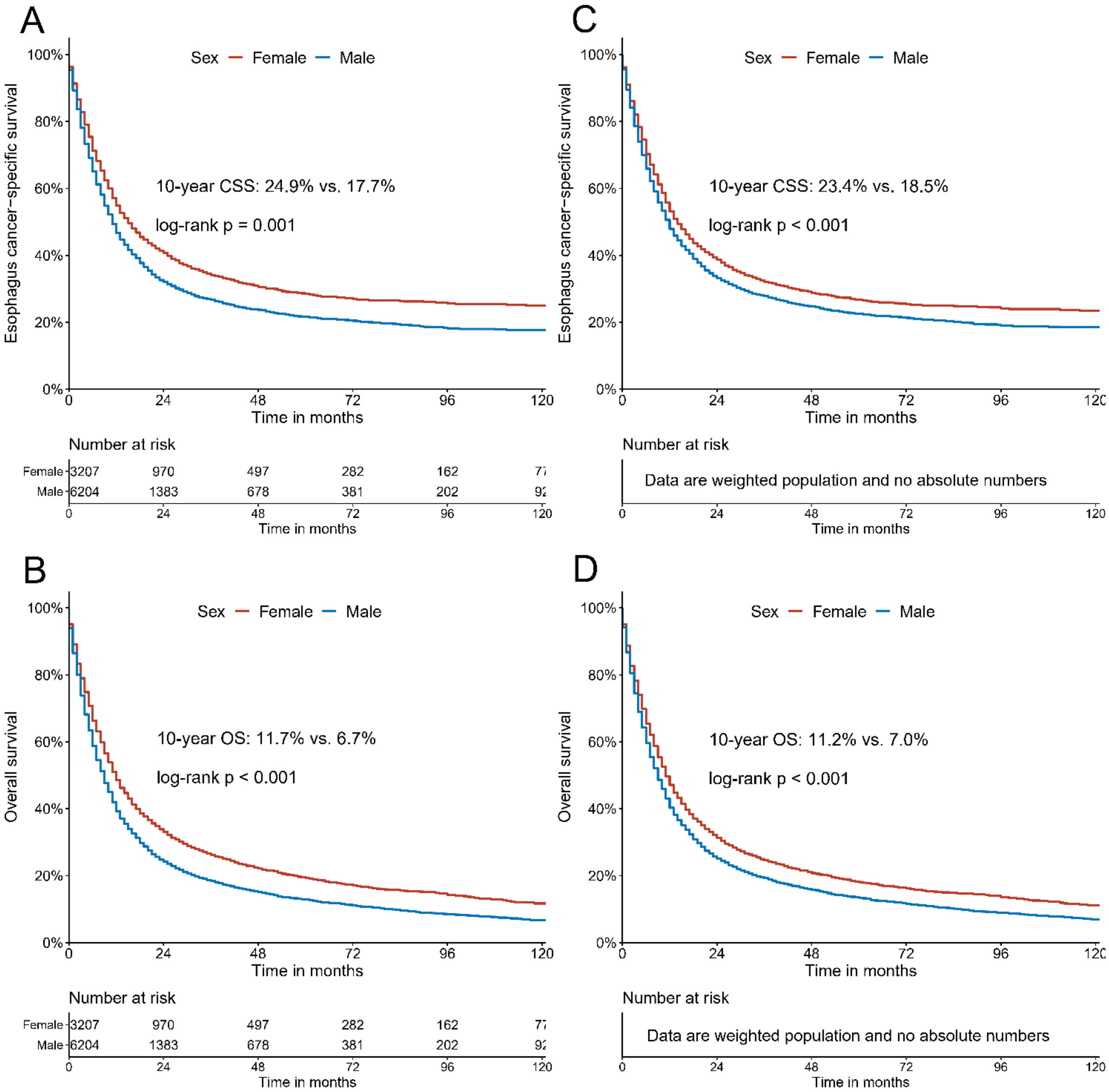

Figure S8. Cancer-specific survival and overall survival curves by sex for patients with esophageal squamous cell carcinoma: (A and B) unadjusted; (C and D) Inverse Probability Weighting-adjusted.

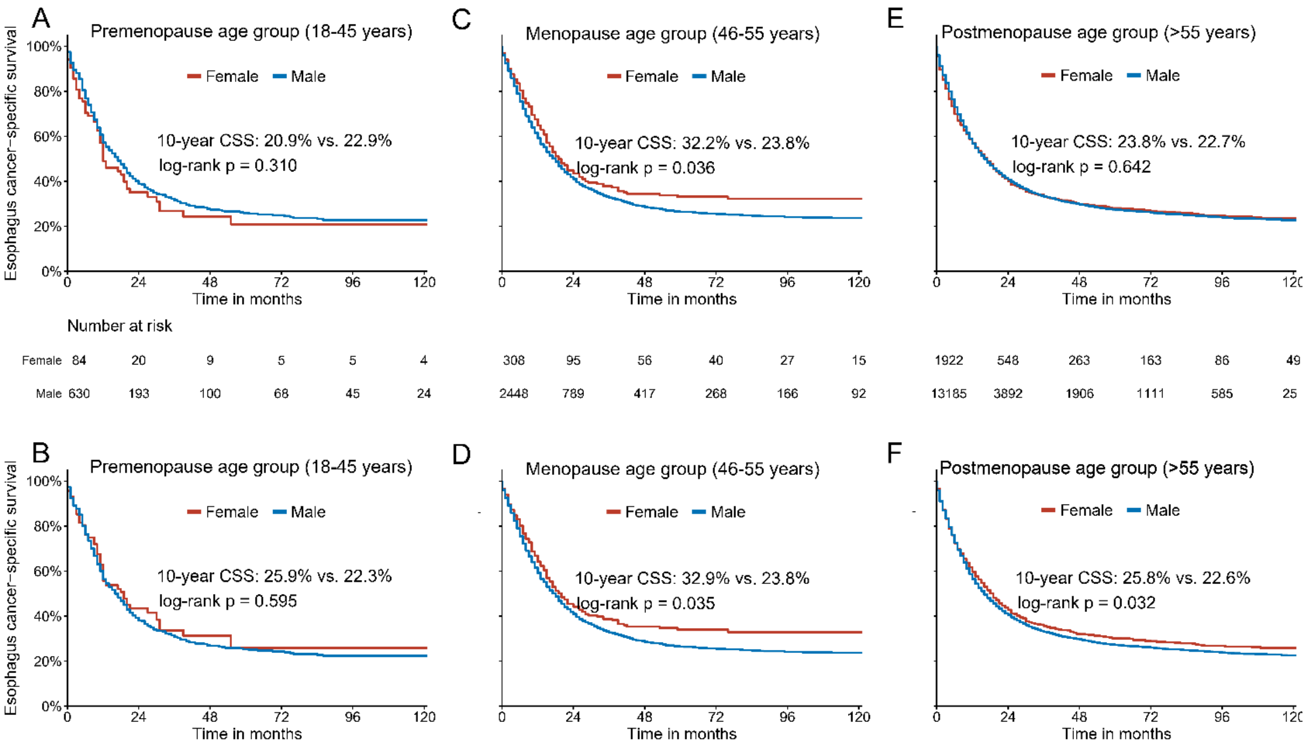

Figure S9. The unadjusted (A, B, C) and Inverse Probability Weighting-adjusted (D, E, F) association between sex and cancer-specific survival in three different age group among patients with esophageal adenocarcinoma (premenopausal age group: age 45 or less; perimenopause age group: age 46-55; and postmenopausal age group: age 56 or more).

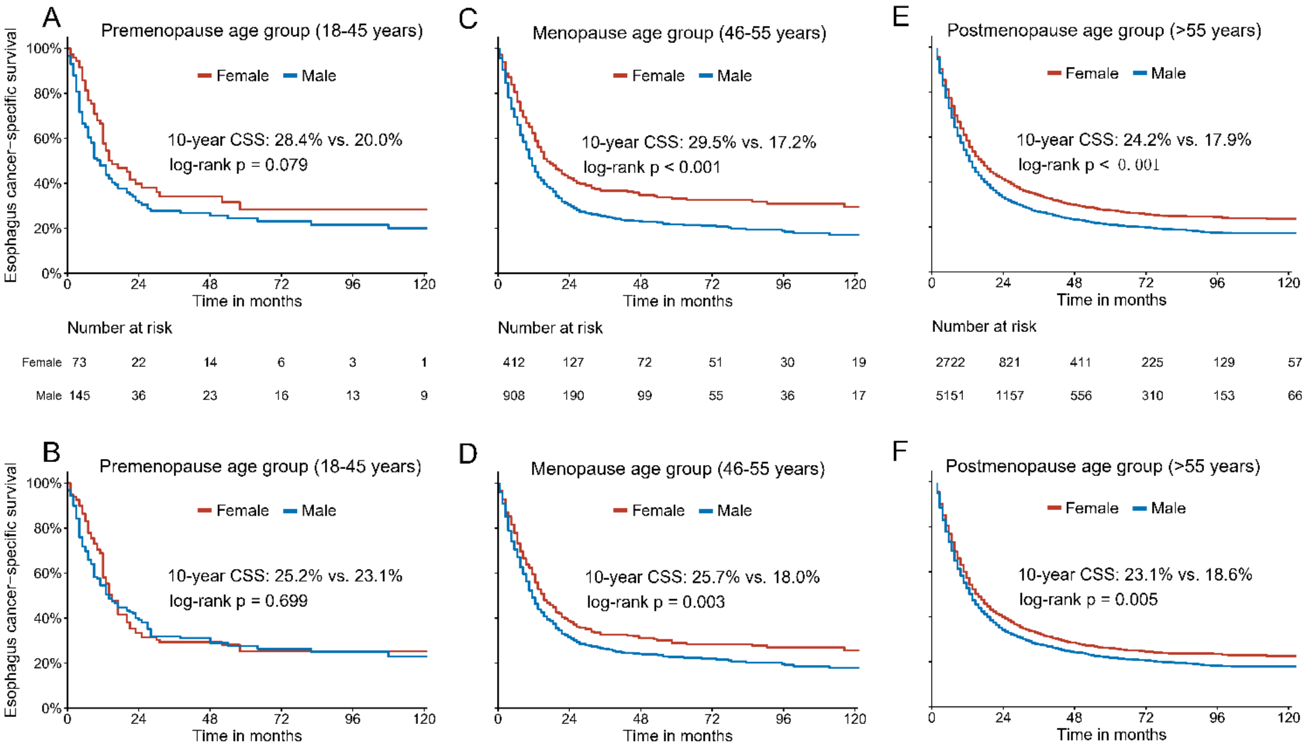

Figure S10. The unadjusted (A, B, C) and Inverse Probability Weighting-adjusted (D, E, F) association between sex and cancer-specific survival in three different age group among patients with esophageal squamous cell carcinoma (premenopausal age group: age 45 or less; perimenopause age group: age 46-55; and postmenopausal age group: age 56 or more).
